# Genomic Analysis of *Laccaria* Genomes at High Altitude

**DOI:** 10.3390/jof11080592

**Published:** 2025-08-14

**Authors:** Yu Bao, Ye Mu, Jinghuan Hu, Mengchao Chen, Jing Xing

**Affiliations:** 1 College of Life Science, Changchun Normal University, Changchun 130032, China; baoy0122@163.com; 2 College of Information Technology, Jilin Agricultural University, Changchun 130118, China; 20231336@mails.jlau.edu.cn (M.C.); 20231274@mails.jlau.edu (J.X.)

**Keywords:** fungi, genome, Tibet, adaptation, high altitude

## Abstract

The Qinghai–Tibet Plateau (QTP) harbors extreme environmental conditions (e.g., low temperature, intense UV radiation, and hypoxia), presenting unique challenges for biological adaptation. However, the genetic mechanisms underlying the adaptation of macrofungi to high-altitude environments on the QTP remain poorly understood. In this study, we de novo sequenced and assembled the genomes of three *Laccaria* species collected from the QTP, aiming to unravel the genomic basis of their adaptation to high altitudes. The genomic data indicates that the genome of high-altitude species is slightly larger than that of their low-altitude relatives, particularly due to LTR retrotransposons, which also show a negative correlation with altitude. The expanded and positively selected gene families in high-altitude species were enriched in pathways related to DNA damage repair, maintenance of cell membrane stability, signal transduction, enzyme activity, stress response, and reproduction. In contrast, contracted gene families in high-altitude species were primarily associated with disease and immune responses, likely due to the reduced pathogen pressure in extreme high-altitude environments. Additionally, species-specific genes of high-altitude *Laccaria* were enriched in functions related to enzyme activity, membrane stability, and signal transduction, further supporting their adaptive roles. Analysis of carbohydrate-active enzymes (CAZymes) showed distinct gene family distributions between high- and low-altitude species, with several families absent in the low-altitude species, suggesting their potential involvement in environmental adaptation. Overall, our findings indicate that genome size expansion driven by LTR retrotransposons, coordinated evolution of gene families, positive selection, and divergence in CAZymes collectively may contribute to the adaptation of *Laccaria* to extreme high-altitude environments. This study provides basic data into the genetic mechanisms of fungal adaptation to harsh plateau environments and lays a foundation for further research on extremophilic fungi.

## 1. Introduction

The Qinghai–Tibet Plateau (QTP) is the highest plateau (average elevation above 4000 m) in the world and is called the roof of the world or the third pole. The QTP mountains reached their current height between the late Miocene and Pliocene periods [1]. The uplift of the altitude has led to unique environmental conditions and diverse species. Conditions on the QTP are characterized by low temperature, low oxygen, reduced presence of pathogens, and strong UV radiation, which together provide a unique environment to study adaptive evolution [2,3]. However, research on fungal species in this area remains limited due to its remote geography, harsh environment, and lack of transportation infrastructure. Fungi are a highly diverse group of heterotrophic eukaryotes characterized by the absence of phagotrophy and the presence of a chitinous cell wall. Armed with these morphological traits and with an extremely high metabolical diversity, fungi have conquered numerous ecological niches and have shaped a whole world of interactions with other living organisms [4]. It is reported that high-altitude lichen individuals showed ecophysiological adaptations to wetter and more shaded conditions. Highly differentiated genome regions contained a number of genes associated with stress response, local environmental adaptation, and sexual reproduction [5]. Pseudogymnoascus is a fungal genus that thrives in cold temperatures and has a preference for temperatures at or below 15 °C. The fungus’s capacity to endure in cold temperatures is attributed to the secondary metabolites it generates [6]. The most striking effect of UV on *F. mangiferae* was developmental-dependent induction of translation related genes [7]. However, research on fungal environmental adaptation has primarily focused on marine fungi, soil fungi, pathogens, and lichens. Studies on genome-wide variations related to environmental adaptation in macrofungi remain entirely uninvestigated.

Our research group has focused on investigating macrofungi in Tibet since 2015, and during that time, we discovered that vegetation in this area is mainly alpine shrubs and meadows, as well as mosses and lichens, especially in areas over 4300 m above sea level. Most of them are species with alpine desert characteristics, and they grow intensively and have small morphologies and strong environmental tolerance. While the macrofungi here show similar characteristics, the elevation has a direct impact on their vertical distribution. The environment over 4300 m is extremely hostile, with the average annual temperature being <0 °C, UV radiation being intense, and wide daily temperature swings. In this regard, we hypothesized that macrofungi there have made a number of evolutionary adaptive changes.

Due to its unique geographical location and climatic environment, the QTP has a wide range of fungi resources. The study of macrofungi in the QTP has mostly focused on its diversity and community composition. The diversity of macrofungi is closely related to the composition of the plant community, temperature, soil type, altitude, and precipitation [8,9]. Previous genome-wide studies on adaptive evolution on the QTP have focused mainly on humans and vertebrates [10,11]. However, to our knowledge, there has been no study on adaptive evolution in terms of a whole-genome analysis.

Because its genome is much smaller than that of plants, fungi are a good model system to study speciation and ecological adaptation in an extreme environment. In a previous study in 2017, we collected 283 macrofungi specimens. According to traditional morphological and molecular biological identification, we found that these comprise 283 macrofungi belonging to 2 phyla, 3 classes, 12 orders, 36 families, 70 genera, and 183 species [12]. We selected the following species of *Laccaria* to study: high-altitude *Laccaria bicolor*, *Laccaria tortilis*, and low-altitude *Laccaria tortilis*, identified as A1, C3, and B2, respectively. In this study, we established the genome-wide data on these macrofungi at different altitudes in the QTP, with the goal of investigating genetic mechanisms by which macrofungi have adapted to the complex extreme conditions on the QTP.

## 2. Materials and Methods

### 2.1. Fungi Material, Genome Sequencing, and Assembly

All of the *Laccaria* mentioned in this study were collected on Shergyla Mountain of QTP with a high altitude of more than 4300 m and a low altitude of ~3000 m. The collected macrofungi specimens were dried at low temperature and then put into a bag with desiccant during transport. The macrofungi collected in Tibet were first preliminarily identified based on their morphological characteristics. Analysis of fungal DNA sequence was used for fungal species identification by the amplification of the internal transcribed spacer region (ITS1-5.8S-ITS2) using PCR [13]. The ITS1-5.8S-ITS2 rDNA was amplified using primers ITS4 (forward primer) and ITS5 (reverse primer) [14]. The identification of the DNA sequence was implemented by comparison of desired DNA sequence data with those available from GenBank database [13]. We selected three *Laccaria* strains distributed at different altitudes as the subjects of this study, namely, *Laccaria bicolor* (A1) and *Laccaria tortilis* (C3) distributed at high altitude (4300 m) and *Laccaria tortilis* (B2) distributed at low altitude. The BLAST (https://blast.ncbi.nlm.nih.gov/Blast.cgi?PROGRAM=blastn&PAGE_TYPE=BlastSearch&LINK_LOC=blasthome (accesseed on 1 March 2025)) search showed that the sequence data of the A1 shared 99% similarity with *Laccaria bicolor* (KM067820.1). B2 and C3 shared 99% similarity with *Laccaria tortilis* (JQ888175.1). Thus, A1 was identified as *Laccaria bicolor*, and B2 and C3 were identified as *Laccaria tortilis*. The sequence data were submitted to Bankit for accession numbers. The GenBank accession numbers of A1, B2, and C3 are PX096150, PX096152, and PX096151.

Genomic DNA was extracted using the Qiagen DNeasy Plant Mini Kit (Qiagen, Germantown, MD, USA) following the manufacturer’s protocol. The harvested DNA was detected by agarose gel electrophoresis and quantified by using Qubit. Whole-genome sequencing was performed on the Illumina HiSeq PE 150 platform. A tailed and ligated to paired-end adaptor and PCR amplified with a 350 bp insert were used for the library construction at Beijing Novogene Bioinformatics Technology Co., Ltd. (Beijing, China). After Illumina PCR adapter reads and low-quality reads from the paired-end were filtered, all good quality paired reads were assembled using the SOAP de novo method [15] (https://sourceforge.net/projects/soapdenovo2/files/SOAPdenovo2/ (accessed on 1 March 2025)) into a number of scaffolds.

### 2.2. Genome Assembly and Annotation

For fungi, we used the program Genewise (Version 2.4.1) [16] to retrieve related coding genes and a complete annotation pipeline, PASA, also as implemented at the Broad Institute. The interspersed repetitive sequences were predicted using the Repeat Masker program [17] (http://www.repeatmasker.org/ (accessed on 1 February 2025)). Tandem repeats were analyzed by using TRF (Version 3.08) [18]. Transfer RNA (tRNA) genes were predicted with tRNAscan-SE (Version 2.0.12) [19]. Ribosome RNA (rRNA) genes were analyzed by using rRNAmmer (Version 1.2) [20]. sRNA, snRNA, and miRNA were predicted by BLAST against the Rfam database (https://rfam.org/, accessed on 1 February 2025) [21,22]. We used six databases to predict gene functions: GO (Gene Ontology) [23], KEGG (Kyoto Encyclopedia of Genes and Genomes) [24,25], KOG (Clusters of Orthologous Groups), NR (Non-Redundant Protein Database) [26], Pfam (http://pfam.xfam.org/ (accessed on 1 February 2025)), and CAZymes (https://www.cazy.org/, accessed on 1 April 2025) (Carbohydrate-Active enzymes Database) [27]. According to the dbcan database [28] using Hmmer (http://hmmer.org/ (accessed on 1 March 2025)), parameter E-value < 1 × 10^−17^, and coverage > 0.45 and using fungi P450 database as the reference database, we identified the coding gene for carbohydrate active enzyme (cazymes).

### 2.3. Gene Family Expansion and Contraction

Expansions and contractions of orthologous gene families were determined using the program CAFÉ (Version 5.0) [29]. This software was used to identify gene families that had undergone expansion and/or contraction. GO enrichment analyses of genes were conducted using web-based agriGO (https://systemsbiology.cau.edu.cn/agriGOv2/index.php (accessed on 1 April 2025)) [30] with the singular enrichment analysis method (SEA).

### 2.4. Positively Selected and Species-Specific Genes

The ratio of non-synonymous substitution per non-synonymous site (Ka) to synonymous substitutions per synonymous site (Ks) is a common way to indicate selective pressure, with Ka/Ks > 1 representing positive selection, Ka/Ks = 1 representing neutral selection, and Ka/Ks < 1 representing purifying selection. For positive selection analyses, we selected low-altitude species with assembled genomes and high-altitude species to identify orthologs. First, we used OrthoMCL (Version 2.0) [31] to identify homologous gene clusters (orthogroups). Multiple sequence alignment was performed for each orthogroup using MUSCLE (Version 5.0) [32] with default parameters. To calculate nonsynonymous (Ka) and synonymous (Ks) substitution rates between pairs of orthogroups, we reverse-translated amino acid alignments to the corresponding codon-based nucleotide alignments using PAL2NAL (Version 14) [33]. The Ka and Ks values were calculated using the GY-HKY method.

We use CD hit software (Version 4.6.1) to cluster protein sequences, setting the screening parameters of identity and alignment length, obtaining the clustering of all protein sequences according to the results of software analysis to identify the species-specific gene, and we used R (Version 3.2.4) for visualization.

## 3. Results and Discussion

### 3.1. Genome Assembly and Annotation

We used the Illumina HiSeq PE150 platform with paired-ends to sequence the entire *Laccaria* genome. A draft de novo assembly of genome size is 109,399,439 bp (A1 *L. bicolor*, high altitude), 104,534,143 bp (B2 *L. tortilis*, low altitude), and 120,365,163 bp (C3 *L. tortilis*, high altitude), consisting of 64,686, 34,057, and 73,143 contiguous overlapping groups, respectively (Table 1 and Table S1). Compared with low-altitude species, high-altitude species had a slightly larger genome size.

A total of 23,719, 25,283, and 27,546 protein-coding genes were predicted (Table 1 and Table S2). In addition to protein-coding genes, various noncoding RNA sequences were identified and annotated, including tRNA, sRNA, snRNA, miRNA, and rRNA (Table S3). We identified and marked 14.6% and 13.5% of the assembly as repeat regions in the high-altitude species, whereas the proportion of low-altitude species was 10.2% (Table 2 and Table S4). In particular, a high proportion of repeat regions were long terminal repeat (LTR) retrotransposons, 27.58% and 27.8% in high-altitude species, whereas 24.15% were in the low-altitude species. The retrotransposon proliferation may be responsible for genome-size expansion in the high-altitude species. In LTR retrotransposons, copia and gypsy families are the most important components; in addition, the gypsy family accounted for a higher proportion in fungi. This is different in plants, and the number of both of them in the high-altitude species is larger than that of their low-altitude relatives (Table S5).

### 3.2. Functional Annotations

**Figure 1:**
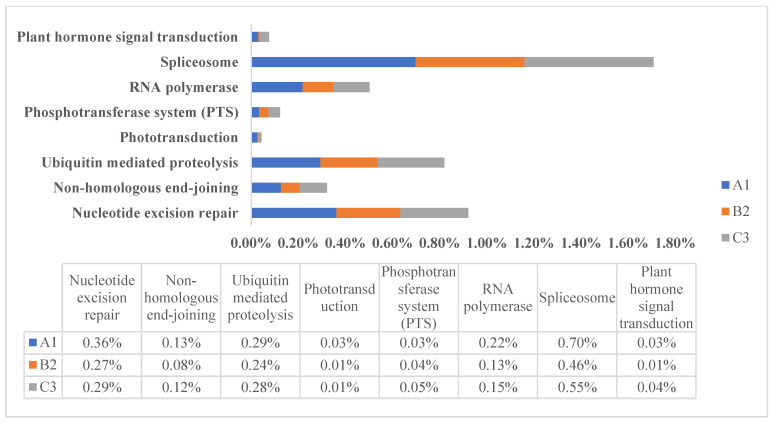
KEGG-related pathways in *Laccaria* genome. The Y-axis represents KEGG pathways, and the X-axis represents the proportion of annotated gene. A1 represents *L. bicolor* of high altitude, B2 represents *L. tortilis* of low altitude, and C3 represents *L. tortilis* of high altitude.

**Figure 2:**
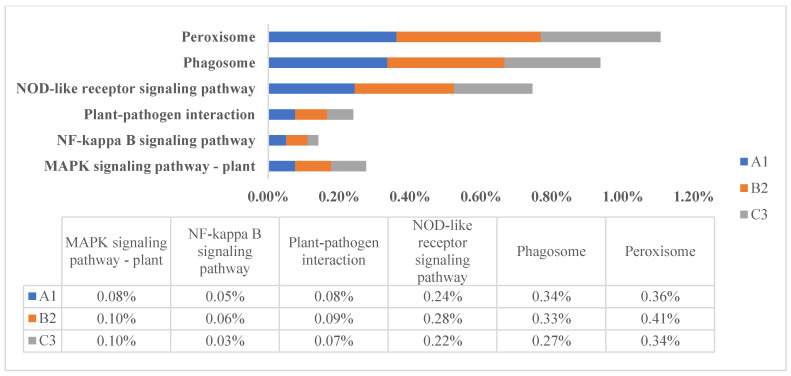
KEGG-related pathways in *Laccaria* genome.

All protein-coding genes were annotated using NR, Pfam, GO, KEGG, and KOG (Table S6). NR has the highest proportion of successful annotations, whereas KOG has the lowest proportion. By analysis of different metabolic pathways in the KEGG database, we found that the gene quantity ratio of high-altitude species is quite different from their low-altitude relatives in some pathways, e.g., pathways of signal transduction, enzyme activity, DNA repair, and photo-morphosynthesis, and the gene quantity ratio of high-altitude species was much larger (Figure 1). It has been reported that ubiquitin-mediated proteolysis impacts almost every aspect of plant growth and development, including plant hormone signal transduction, reproduction, abiotic stress responses, and photomorphogenesis [34]. Also, it has been reported that the convergence points among hormone signal transduction cascades are considered as cross-talk, which is crucial for plant development, as well as for plant responses to biotic and abiotic stresses [35]. Spliceosomes, as basal regulatory factors (distinct from direct stress effectors), may optimize transcriptome plasticity under environmental constraints through their genomic variations [36]. Such multifunctional biological processes are of even greater importance for high-altitude fungi in the harsh environment on the QTP, which may be related to environmental adaptability. While in some pathways related to disease and immune response, such as the NOD-like receptor signaling pathway, the NF-κappaB signaling pathway, and the MAPK signaling pathway, high-altitude species have a smaller gene quantity ratio (Figure 2). The NOD-like receptor (NLR) plays an important role in recognition of intracellular ligands. The NLR family contains a variety of proteins, which are responsible for identifying specific pathogens or host-derived damage signals in the cytoplasm and triggering innate immune responses. Once activated, NLR can activate a series of signaling pathways, including the NF-κappaB signaling pathway, the MAPK signaling pathway, and apoptosis.

### 3.3. Gene Family Expansion and Contraction

Significant expansion or contraction in the size of particular gene families is often associated with adaptive evolution of a species [37,38]. In this study, the selection of low-altitude *Laccaria* and *Agaricus bisporus* as reference sequences ensures the reliability of identifying adaptive gene families, primarily based on the following scientific rationales: (1) Low-altitude *Laccaria* provides an intra-genus genetic variation baseline, enabling precise identification of altitudinal adaptation-driven expansions and contractions in gene families. (2) *Agaricus bisporus*, as the model species of *Agaricales* and the first fully sequenced edible fungus with optimal assembly quality, establishes a definitive core gene set to filter out non-specific gene family evolution. Comparisons of genomes of low-altitude *Laccaria* (B2 *L. tortilis*) and *Agaricus bisporus* identified a total 364 and 94 gene families that are expanded in high-altitude fungi (A1 *L. bicolor* and C3 *L. tortilis*) and 122 and 134 gene families that are contracted (Table 3). Based on Kyoto Encyclopedia of Genes and Genomes (KEGG) and Gene Ontology (GO) annotations, expanded gene families are significantly enriched in pathways related to DNA damage repair, maintenance of cell membrane stability, signal transduction, enzyme activity, response to stress, and reproduction (Table 4, Table 5, Table 6 and Table 7). The extremely intense UV radiation on the QTP may influence plant growth and developmental processes, and in particular, it can cause DNA, RNA, and protein synthesis damage [39,40]. Existing studies have demonstrated that plateau plants have evolved a suite of mechanisms to adapt to high-altitude environments. Dramatic contractions and expansions of gene families were likely key mechanisms for *Crucihilaya himalaica* to adapt to highland environments [41]. Specific SINE transposons promote adaptation of *Prunus* species to high altitudes by enhancing beneficial metabolites [42]. However, genomic variations underlying high-altitude adaptation in macrofungi have not been documented to date. The expanded gene families of high-altitude species are involved in base excision repair, suggesting that *L. bicolor* at high-altitude has improved DNA repair system to adapt to the harsh habitats of strong UV radiation. The enrichment of the DNA repair pathway also maintains the stability of the genome. Moreover, high-UV-B radiation is a common stress that both animals and plants on the QTP must cope with. The DNA repair and radiation responses pathways have also played important roles in highland adaptation in the Tibetan hot-spring snake [43], Tibetan antelope [44], Tibetan highland barley [45], and Tibetan chicken [46]. With the increase in altitude and decrease in temperature in the QTP, enzyme activity is generally reduced, and the catalytic efficiency of a given enzyme is affected. The enrichment of enzyme activity is also crucial for adaptation, which is consistent with a report that SOD activity of *Melilotoides ruthenicus* increases under low temperature stress [47]. Another important effect of low-temperature on organisms is the change in the structure of their cell membranes, which goes from liquid crystal to gel phase. The gel phase reduces energy supply and accumulates toxic metabolites. Maintenance of cell membrane stability may be important for better survival in the harsh environment of the QTP.

Based on KEGG and GO annotations, we noted that there are significantly contracted gene families in the *L. bicolor* genome of high altitude. This may be functionally related to disease and immune responses, such as the Toll-like receptor signaling pathway and plant–pathogen interactions. Due to low temperature, drought, and high UV radiation, it is possible that there are relatively few microorganisms on the QTP, such that bacterial pathogen infection is relatively low [48]. The Toll receptor is an N-terminal component of the nucleotide-binding site (NBS) disease resistance protein family. Most bacteria interact with Toll-like receptors on the surface of the host cell membrane to stimulate the NF-κappaB signaling pathway during immune responses [47]. Expansion or contraction of gene families at high altitude may be a fundamental strategy for fungi to adapt to the harsh environments on the QTP.

### 3.4. Positive Selection in Single-Copy Genes

**Figure 3:**
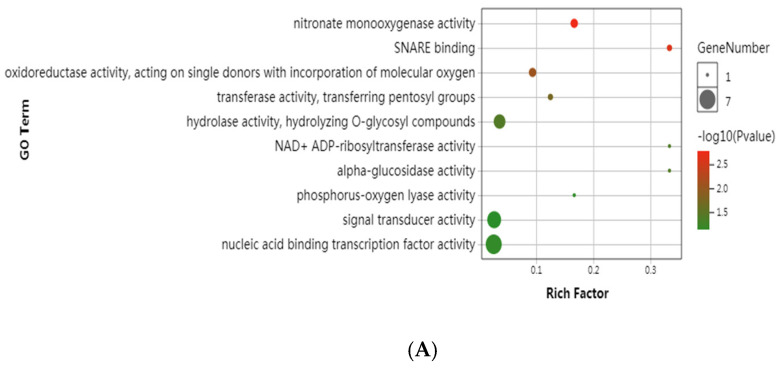

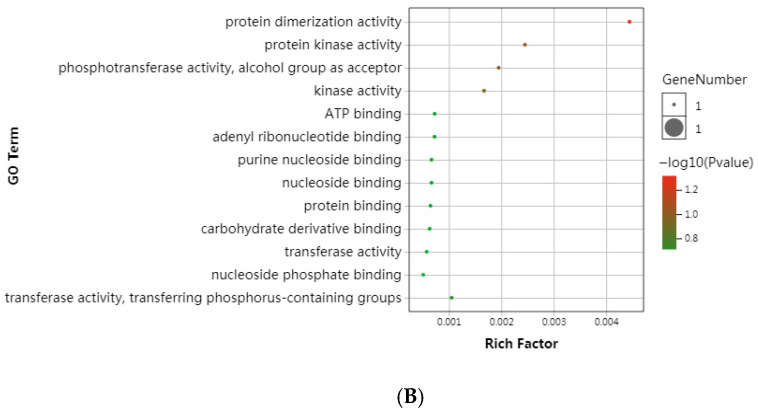
Positive selection genes in *Laccaria* genome. (**A**) Positive selection genes in the A1 (*L. bicolor*, high altitude) genome. (**B**) Positive selection genes in the C3 (*L. tortilis*, high altitude) genome.

Orthologs that show signs of positive selection usually undergo adaptive divergence [49]. In some specific genes, the increasing rate of nonsynonymous substitution (KA) compared with synonymous substitution (KS) may reveal adaptive evolution of organisms on the molecular level [50]. In this study, we conducted a positive selection analysis using the genomic sequences of high-altitude *Laccaria* and their low-altitude relatives: A1 (*L. bicolor* of high altitude) vs. B2 (*L. tortilis* of low altitude) and C3 (*L. tortilis* of high altitude) vs. B2 (*L. tortilis* of low altitude). For comparative analyses of genomes, there are 2596 single-copy orthologous genes in A1 vs. B2 and 3602 single-copy orthologous genes in C3 vs. B2. Among these genes, 22 and 16 genes are under positive selection (PSG, ka/ks > 1) in high-altitude fungi, A1 and C3, respectively. A GO functional classification of the PSGs in the high-altitude *Laccaria* genome shows that there are several categories associated with pathways related to signal transduction and enzyme activity (Figure 3). It is notable that significantly expanded gene families and PSGs are both enriched in signal transduction and enzyme activities, which is again proof that gene expansion and positive selection are important mechanisms of adaptive evolution in this biological environment.

Simultaneously, we observed a highly intriguing phenomenon: the high-altitude populations of *Laccaria bicolor* and *Laccaria tortilis* possess distinct sets of positively selected genes and enriched pathways. The underlying reasons for this are likely the result of multifaceted complexity acting in concert. Biological systems often possess multiple molecular pathways to address identical environmental challenges, such as high-altitude stress. Consequently, these two species may have evolved divergent sets of genes or regulatory changes, appearing to take different evolutionary paths. Yet, ultimately, they converge on enhancing the same core physiological functions, such as cold resistance and antioxidant capacity. Although closely related, these sister species have accumulated unique genetic foundations during their long-term divergence. This distinctiveness manifests in differences in gene copy number, regulatory network architecture, and gene–gene interaction patterns. Furthermore, natural selection is not the sole driver of evolution; stochastic factors, such as genetic drift, may also influence the “selection bias” observed in the positively selected genes. This fascinating phenomenon underscores the potential for closely related species to adopt divergent molecular evolutionary trajectories in adapting to similar selective pressures. It clearly warrants further in-depth investigation.

### 3.5. Species-Specific Gene of High-Altitude Adaptation

**Figure 4:**
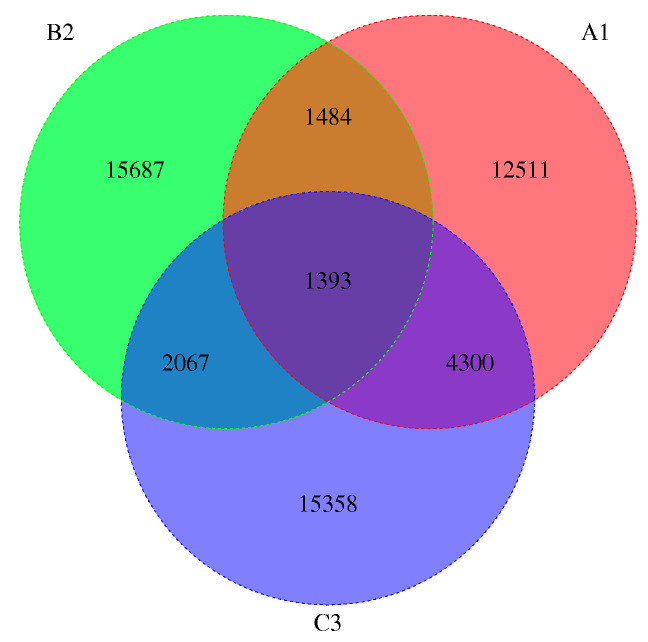
Venn diagram showing unique and shared genes in the *Laccaria* genome. A1 represents *L. bicolor* of high altitude, B2 represents *L. tortilis* of low altitude, and C3 represents *L. tortilis* of high altitude.

It is possible that core genes and specific genes between different species correspond with commonness and characteristics. Based on whole-genome sequencing, we obtained the following data through analysis of protein clustering results. Based on the statistical analysis of shared genes across three samples, we found that A1 (*L. bicolor*, high altitude) and B2 (*L. tortilis*, low altitude) shared 2877 genes, and B2 (*L. tortilis*, low altitude) and C3 (*L. tortilis*, high altitude) shared 3460 genes. This may be attributed to both B2 and C3 being *L. tortilis*. Notably, A1C3 exhibited a higher number of shared genes (5693), exceeding those of B2C3. We speculate this may result from both A1 and C3 being high-altitude species. However, further experimental validation is required to (1) identify which of these shared genes are associated with environmental adaptation, (2) determine potential deterministic genetic factors, and (3) elucidate their functional mechanisms. We pay special attention to the specific gene of high-altitude species and their enriched pathways. The number of core genes of *Laccaria* is 1393, and the high-altitude *Laccaria* have 12,511 and 15,358 specific genes, respectively (A1 and C3) (Figure 4). According to GO annotation, the specific genes of high-altitude *Laccaria* are mainly enriched in pathways related to enzyme activity, maintenance of cell membrane stability, reproduction, photomorphogenesis, and signal transduction (Table 8 and Table 9).

### 3.6. CAZymes Gene of High-Altitude Adaptation

**Figure 5:**
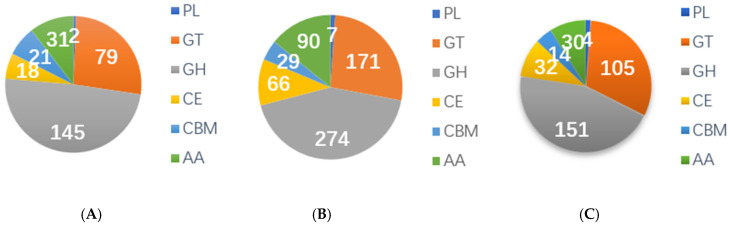
Carbohydrate-related enzymes in CAZymes in *Laccaria* genome. (**A**) CAZymes gene in A1 (*L. bicolor* of high altitude), (**B**) CAZymes gene in B2 (*L. tortilis* of low altitude), and (**C**) CAZymes gene in C3 (*L. tortilis* of high altitude). PL—polysaccharide lyases; GT—glycosyl transferases; GH—glycoside hydrolases; CE—carbohydrate esterases; CBM—carbohydrate-binding module; AA—auxiliary activities.

In fungi, cazymes contain some important enzymes involved in hydrolysis and synthesis of complex carbohydrates [51,52]. The CAZymes database describes the structure-related enzyme catalysis or carbohydrate functional domain modules, in which gene function involves hydrolysis, modification, transfer, breaking glycoside bonds, etc., which play an important role in the normal growth and development of fungi [52]. Based on the CAZymes statistical analysis of *Laccaria*, we found that the proportion of glycoside hydrolases (GHs) is the largest (Figure 5). Glycoside hydrolases (GHs) are a group of enzymes capable of hydrolyzing glycosidic bonds, playing a role in the processes of sugar hydrolysis and synthesis in organisms. Existing studies have shown that GH genes are differentially expressed during plant development and under abiotic stress, revealing their potential roles in plant development and stress responses [53,54]. Concurrently, studies have also reported that GH family members play critical roles in vital fungal functions including cell survival, stress response, virulence, and even immune priming [55].

Comparing the carbohydrate enzyme gene families of high-altitude fungi and their low-altitude fungi, the number of genes is quite different. It is worth noting that some gene families are absent in low-altitude fungi, such as GH9, GH6, GH4, GH16, CE7, CE5, and GT32 (Table S7). This may be related to the adaptability of fungi at different altitudes due to their environments, but this conclusion requires further gene expression analysis for verification.

We observed an intriguing phenomenon: among the three materials, *L. tortilis* at low altitude exhibited greater CAZyme diversity than both *L. bicolor* and *L. tortilis* at high altitudes. This pattern aligns with findings in marine fungi, where the relative proportion of secretory CAZymes increases with depth, a trend similarly observed in prokaryotes [56]. This may be attributed to decreased enzyme activity resulting from elevated altitude and concomitant temperature reduction. Comparing the high-altitude species *L. bicolor* and *L. tortilis*, we observed lower CAZyme diversity in *L. bicolor* than in *L. tortilis*. Does this indicate that *L. tortilis* is better adapted to high-altitude environments than *L. bicolor*? This hypothesis warrants further investigation through transcriptomic and metabolomic analyses.

## 4. Conclusions

Organisms that live on the QTP face a variety of abiotic stress under the harsh environmental conditions, including low temperature, extremely strong UV-B radiation, and low oxygen levels. In this study, we performed de novo genome assemblies for *Laccaria*: two high-altitude species (*L. bicolor* and *L. tortilis*) and one low-altitude species (*L. tortilis*). The genome size of macrofungi at different altitudes is different, with the genome of high-altitude species being somewhat larger than that of their low-altitude relatives. Moreover, repeat elements, especially the LTR retrotransposons, are negatively correlated with altitude. Proliferation of LTR retrotransposons may at least partly be responsible for the increased genome size of high-altitude *Laccaria*.

High-altitude fungi have more genes in pathways related to signal transduction, enzyme activity, DNA repair, and photomorphosynthesis. Compared with low-altitude fungi, gene families of the high-altitude fungi that underwent significant expansion and are species-specific are enriched in pathways related to DNA damage repair, maintenance of cell membrane stability, signal transduction, enzyme activity, response to stress, and reproduction, whereas the contraction of gene families is related to defense pathogens. The positive selected gene families in the high-altitude *Laccaria* genome showed that there are several categories associated with pathways related to signal transduction and enzyme activity. This supports the idea that gene expansion, positive selection, and species-specific genes are important for adaptive evolution in this biologically harsh environment.

The evolution of carbohydrate enzyme genes in different gene families has also revealed the environmental adaptability of high-altitude fungi, in particular that some gene families are absent in low-altitude fungi. This, in turn, may be related to environmental adaptability. However, we must emphasize that the study focuses primarily on genomic analysis and does not fully address the physiological, biochemical, or ecological aspects of fungal adaptation. Also, it is necessary to investigate gene expression under different environmental conditions and to explore the interactions between *Laccaria* species and other organisms (e.g., plants and bacteria) on the Qinghai–Tibet Plateau to better understand how these species respond to dynamic stressors. This will be the focus of our subsequent research. While genomic analysis provides evolutionary insights, functional validation requires transcriptomics. Our sampling across 4300 m elevation gradients prevented RNA preservation due to (i) lack of power sources for cryopreservation equipment and (ii) delayed sample transit (>72 h) to sequencing facilities. Overall, this study has provided some basic information on macrofungi in the alpine tundra of Tibet. The genome analysis of macrofungi at different altitudes has revealed the possible adaptable genetic changes of macrofungi at high altitudes following millions of years of evolution.

## Figures and Tables

**Table 1:** Genome assembly features of *Laccaria*.

Sp.	A1 (High Altitude) (*L. bicolor*)	B2 (Low Altitude) (*L. tortilis*)	C3 (High Altitude) (*L. tortilis*)
Genome size (bp)	109,399,439	104,534,143	120,365,163
No. of contigs	64,686	34,057	73,143
GC content (%)	47.03	47.79	47.66
No. of scaffolds	63,946	33,829	72,483
GC content (%)	47.03	47.79	47.66
N50 contig length (bp)	2245	6000	2096
N50 scaffold length (bp)	2264	6031	2109
N90 contig length (bp)	755	1158	767
N90 scaffold length (bp)	762	1164	773
No. of protein-coding genes	23,719	25,283	27,546

**Table 2:** Repeat sequences in *Laccaria* genome.

Sp.	No. of Repeat (in Genome%)	No. of Repbase (in Genome%)	No. of TRF (in Genome%)	No. LTR (in Genome%)	No. LTR (in Repeat%)
A1 (*L. bicolor*, high altitude)					
	115,354 (14.6)	37,284 (8.6)	78,070 (6.0)	31,810 (7.96)	27.58%
B2 (*L. tortilis*, low altitude)					
	80,603 (10.2)	23,862 (5.2)	56,741 (5.0)	19,464 (4.67)	24.15
C3 (*L. tortilis*, high altitude)					
	115,278 (13.5)	37,938 (8.0)	77,340 (5.4)	32,046 (7.41)	27.8

**Table 3:** Statistics of the number of expanded/contract gene families of *Laccaria*.

Sp.	A1 *L. bicolor* High-Altitude	C3 *L. tortilis* High-Altitude
No. of expansion gene family	364	94
No. of contraction gene family	122	134

**Table 4:** GO function annotation of the significantly expansive and contract gene families in A1 (*L. bicolor*, high altitude).

	GO Term	Gene Ratio	Bg Ratio	*p*-Value	Rich Factor
Expand	Translational termination	5/2503	13/31,381	0.002	4.82
Gene Families	Proton-transporting two-sector ATPase complex, catalytic domain	6/2503	16/31,381	0.001	4.70
	Vesicle coat	5/2503	20/31,381	0.018	3.13
	Cytoplasmic vesicle membrane	5/2503	20/31,381	0.018	3.13
	Transcription from RNA polymerase III promoter	5/2503	21/31,381	0.022	2.99
	Actin binding	7/2503	30/31,381	0.008	2.93
	RNA polymerase II transcription factor activity, sequence-specific DNA binding	5/2503	22/31,381	0.027	2.85
	Membrane coat	13/2503	66/31,381	0.002	2.47
	Asexual reproduction	6/2503	32/31,381	0.039	2.35
	Oxidoreductase activity, acting on the CH-CH group of donors, NAD or NADP as acceptor	8/2503	49/31,381	0.039	2.05
Contract	Helicase activity	5/70	425/31,381	0.003	5.27
Gene Families	Transporter activity	5/70	648/31,381	0.018	3.46

**Table 5:** KEGG function annotation of the significantly expansive and contract gene families in A1 (*L. bicolor*, high altitude).

	KEGG Term	Gene Ratio	Bg Ratio	*p*-Value	Rich Factor
Expand gene families	Amino acid metabolism	4/114	33/3198	0.03	3.40
	Signal transduction	2/114	8/3198	0.01	7.00
Contract gene families	Plant–pathogen interaction	1/3	27/2038	0.03	50.30
	Toll-like receptor signaling pathway	1/3	21/2038	0.01	32.30
	One carbon pool by folate	1/3	17/2038	0.02	40.00

**Table 6:** GO function annotation of the significantly expansive and contract gene families in C3 (*L. tortilis*, high altitude).

	GO Term	Gene Ratio	Bg Ratio	*p*- Value	Rich Factor
Expand	Antiporter activity	5/1474	35/45,484	0.005	4.41
gene families	Water-soluble vitamin biosynthetic process	7/1474	67/45,484	0.006	3.22
	Vitamin biosynthetic process	7/1474	67/45,484	0.006	3.22
	Secondary active transmembrane transporter activity	5/1474	59/45,484	0.042	2.62
	Response to stress	7/1474	66/45,484	9.90 × 10^−10^	3.27
	Coenzyme biosynthetic process	6/1474	76/45,484	0.037	2.44
	Base excision repair	11/1471	176/45,484	0.03	2.00
Contract	DNA integration	10/416	431/45,484	0.008	2.54
gene families	DNA binding	26/416	1814/45,484	0.022	1.57
	Cellular aromatic compound metabolic process	55/416	4661/45,484	0.047	1.29

**Table 7:** KEGG function annotation of the significantly expansive and contract gene families in C3 (*L. tortilis*, high altitude).

	KEGG Term	Gene Ratio	Bg Ratio	*p*- Value	Rich Factor
Expand gene families	Amino acid metabolism	4/114	33/3198	0.03	3.40
	Signal transduction	2/114	8/3198	0.01	7.00
Contract gene families	Galactose metabolism	2/31	32/3198	0.037663	6.45
	Proteasome	2/31	32/3198	0.037663	6.45
	Inositol phosphate metabolism	2/31	36/3198	0.046711	5.73
	Citrate cycle (TCA cycle)	3/31	64/3198	0.023059	4.83
	Carbon metabolism	2/31	265/3198	0.011524	2.72

**Table 8:** GO function annotation of the specific gene in A1 (*L. bicolor*, high altitude).

GO-Id	GO Term	Gene Ratio	Bg Ratio	*p* -Value	Rich Factor
GO: 0000123	Histone acetyltransferase complex	10/18,185	26/127,883	2.00 × 10^−3^	2.70
GO: 0071805	Potassium ion transmembrane transport	5/18,185	13/127,883	2.80 × 10^−2^	2.70
GO: 0080134	Regulation of response to stress	6/18,185	16/127,883	1.80 × 10^−2^	2.64
GO: 0019888	Protein phosphatase regulator activity	17/18,185	66/127,883	9.60 × 10^−3^	1.81
GO: 0051607	Defense response to virus	10/18,185	39/127,883	4.30 × 10^−2^	1.80
GO: 0019899	Enzyme binding	30/18,185	134/127,883	7.20 × 10^−3^	1.57
GO: 0000003	Reproduction	73/18,185	356/127,883	7.80 × 10^−4^	1.44
GO: 0016310	Phosphorylation	199/18,185	1175/127,883	5.30 × 10^−3^	1.19
GO: 0098796	Membrane protein complex	146/18,185	894/127,883	4.20 × 10^−2^	1.15
GO: 0016301	Kinase activity	356/18,185	2276/127,883	3.00 × 10^−2^	1.10

**Table 9:** GO function annotation of the specific gene in C3 (*L. tortilis*, high altitude).

GO-Id	GO Term	Gene Ratio	Bg Ratio	*p*-Value	Rich Factor
GO: 0030076	Light-harvesting complex	6/28,632	9/127,883	5.60 × 10^−3^	2.97
GO: 0006744	Ubiquinone biosynthetic process	7/28,632	12/127,883	7.60 × 10^−3^	2.61
GO: 0071555	Cell wall organization	11/28,632	22/127,883	4.10 × 10^−3^	2.23
GO: 0098533	ATPase dependent transmembrane transport complex	19/28,632	51/127,883	1.20 × 10^−2^	1.66
GO: 0001510	RNA methylation	31/28,632	92/127,883	8.60 × 10^−3^	1.5
GO: 0016407	Acetyltransferase activity	81/28,632	243/127,883	6.20 × 10^−5^	1.49
GO: 0015171	Amino acid transmembrane transporter activity	37/28,632	111/127,883	5.40 × 10^−3^	1.49
GO: 0016775	Phosphotransferase activity, nitrogenous group as acceptor	120/28,632	366/127,883	3.30 × 10^−6^	1.46
GO: 0019954	Asexual reproduction	38/28,632	125/127,883	2.30 × 10^−2^	1.36
GO: 0038023	Signaling receptor activity	161/28,632	566/127,883	4.60 × 10^−4^	1.27

## Data Availability

The original contributions presented in this study are included in the article/Supplementary Material. Further inquiries can be directed to the corresponding author.

## Supplementary Materials

The following supporting information can be downloaded at https://www.mdpi.com/article/10.3390/jof11080592/s1, Table S1: Statistics of characteristics of *Laccaria* genome; Table S2: Statistics of predicted protein-coding genes in *Laccaria*; Table S3: Statistics of noncoding RNA in *Laccaria* genome; Table S4: Repeat sequences (repbase, trf) in *Laccaria* genome; Table S5: Statistics of LTR in *Laccaria* genome; Table S6: Information of function annotation in *Laccaria* genome; Table S7: CAZymes in *Laccaria* genome.

## Abbreviations

QTP	Qinghai–Tibet Plateau
PCR	Polymerase chain reaction
LTR	Long terminal repeat
DNA	Deoxyribonucleic acid
PASA	Primer-aligned sequence analysis
RNA	Ribonucleic acid
CAZymes	Carbohydrate-active enzymes
KEGG	Kyoto Encyclopedia of Genes and Genomes
KOG	Clusters of orthologous groups
NR	Non-Redundant Protein Database
SEA	Singular enrichment analysis
GO	Gene Ontology
MAPK	Mitogen-Activated Protein Kinase
NLR	NOD-like receptor
GHs	Glycoside hydrolases
