# Supplementary material for: Genomic Analysis of Laccaria Genomes at High Altitude
Source: J Fungi (Basel). 2025 Aug 14;11(8):592. doi: 10.3390/jof11080592 (PMC12387768; doi:10.3390/jof11080592)
Supplement: Supplementary file 1 [file jof-11-00592-s001.zip › jof-3768217-supplementary.pdf]

**Table S1.** Statistics of characteristics of *Laccaria* genome.

| Sp. | Total Num(>500bp) | Total Length(bp) | N50 Length(bp) | N90 Length(bp) | Max Length(bp) | Min Length(bp) | Sequence GC% |
|---|---|---|---|---|---|---|---|
| contig | | | | | | | |
| A1 | 64,686 | 109,389,279 | 2,245 | 755 | 393,830 | 200 | 47.03 |
| B2 | 34,057 | 104,531,380 | 6,000 | 1,158 | 93,338 | 201 | 47.79 |
| C3 | 73,143 | 120,356,889 | 2,096 | 767 | 118,576 | 200 | 47.66 |
| | Scaffold | | | | | | |
| A1 | 63,946 | 109,399,439 | 2,264 | 762 | 393,830 | 500 | 47.03 |
| B2 | 33,829 | 104,534,143 | 6,031 | 1,164 | 93,338 | 500 | 47.79 |
| C3 | 72,483 | 120,365,163 | 2,109 | 773 | 118,576 | 500 | 47.66 |

**Table S2.** Statistics of predicted protein-coding genes in *Laccaria*.

| Sp. | A1 | B2 | C3 |
|---|---|---|---|
| Gene Number: | 23,719 | 25,283 | 27,546 |
| Gene Length: | 20,548,929 | 27,827,062 | 24,080,648 |
| GC Content: | 50.96 | 53.32 | 53.43 |
| % of Genome (Genes): | 18.78 | 26.62 | 20.01 |
| Gene Average Length: | 866 | 1,101 | 874 |
| Gene Internal Length: | 88,850,510 | 76,707,081 | 96,284,515 |
| Gene Internal GC Content: | 46.12 | 45.78 | 46.22 |
| % of Genome (internal): | 81.22 | 73.38 | 79.99 |

**Table S3.** Statistics of noncoding RNA in *Laccaria* genome.

| | A1(*L. bicolor,* high-altitude) | | | | |
|---|---|---|---|---|---|
| **Type** | | **Number** | **Average length(bp)** | **Total length(bp)** | **% in Genome** |
| tRNA | | 445 | 87 | 38,883 | 0.035542% |
| sRNA | | 1 | 43 | 43 | 0.000039% |
| snRNA | | 30 | 104 | 3,145 | 0.002875% |
| miRNA | | 3 | 221 | 663 | 0.000606% |
| rRNA | rRNA | 8 | 917 | 7339 | 0.006708% |
| | 5s (de-novo) | 4 | 112 | 446 | 0.000408% |
| | 5.8s (de-novo) | 0 | 0 | 0 | 0.000000% |
| | 18s (de-novo) | 3 | 1283 | 3,849 | 0.003518% |
| | 28s (den-ovo) | 1 | 3044 | 3,044 | 0.002782% |
| | B2 (*L. tortilis,* low-altitude) | | | | |
| Type | | Number | Average length(bp) | Total length(bp) | % in Genome |
| tRNA | | 598 | 87 | 52,081 | 0.049822% |
| sRNA | | 2 | 110 | 220 | 0.000210% |
| snRNA | | 40 | 125 | 5,039 | 0.004820% |
| miRNA | | 5 | 183 | 919 | 0.000879% |
| rRNA | rRNA | 13 | 924 | 12011 | 0.011490% |
| | 5s (de-novo) | 7 | 110 | 772 | 0.000739% |
| | 5.8s (de-novo) | 0 | 0 | 0 | 0.000000% |
| | 18s (de-novo) | 4 | 1422 | 5,690 | 0.005443% |
| | 28s (de-novo) | 2 | 2774 | 5,549 | 0.005308% |
| | C3 (*L. tortilis,* high-altitude) | | | | |
| Type | | Number | Average length(bp) | Total length(bp) | % in Genome |
| tRNA | | 550 | 86 | 47,542 | 0.039498% |
| sRNA | | 23 | 136 | 3,131 | 0.002601% |
| snRNA | | 32 | 124 | 3,998 | 0.003322% |

| | | | | | |
|---|---|---|---|---|---|
| miRNA | | 2 | 146 | 293 | 0.000243% |
| rRNA | rRNA | 10 | 810 | 8103 | 0.006732% |
| | 5s (de-novo) | 7 | 113 | 790 | 0.000656% |
| | 5.8s (de-novo) | 0 | 0 | 0 | 0.000000% |
| | 18s (de-novo) | 1 | 1508 | 1,508 | 0.001253% |
| | 28s (de-novo) | 2 | 2902 | 5,805 | 0.004823% |

**Table S4.** Repeat sequences (repbase、trf) in *Laccaria* genome.

| **Sp.** | **A1** | **B2** | **C3** |
|---|---|---|---|
| Repbase | (*L. bicolor*, high-altitude) | (*L. tortilis*, low-altitude) | (*L. tortilis*, high-altitude) |
| No.LTR (In genome%) | 31810 (7.96) | 19464 (4.67) | 32046 (7.41) |
| No.DNA (In genome%) | 3382 (0.42) | 2719 (0.37) | 3652 (0.40) |
| No.LINE (In genome%) | 1534 (0.13) | 1182 (0.08) | 1626 (0.12) |
| No.SINE (In genome%) | 63 (0.003) | 73 (0.004) | 69 (0.003) |
| No.RC (In genome%) | 471 (0.12) | 386 (0.10) | 512 (0.12) |
| No.Unknown (In genome%) | 24 (0.001) | 38 (0.002) | 33 (0.002) |
| Trf | | | |
| No.TR (In genome%) | 44609 (4.0726) | 32372 (3.5412) | 44392 (3.6758) |
| NO.Minisatellite DNA (In genome%) | 32817 (1.9474) | 23638 (1.4423) | 32275 (1.7579) |
| No.Microsatellite DNA (In genome%) | 644 (0.0283) | 731 (0.0436) | 673 (0.0264) |

**Table S5.** Statistics of LTR in *Laccaria* genome.

| **Sp.** | **A1(*L. bicolor*) high-altitude** | **B2 (*L. tortilis*) low-altitude** | **C3 (*L. tortilis*) high-altitude** |
|---|---|---|---|
| LTR/Gypsy (%In LTR) | 25942 (81.55) | 15982 (82.11) | 26057 (81.31) |
| LTR/Pao (%In LTR) | 467 (1.47) | 401 (2.06) | 485 (1.51) |
| LTR/ERVK (%In LTR) | 133 (0.42) | 174 (0.89) | 131 (0.41) |
| LTR/Ngaro (%In LTR) | 378 (1.19) | 104 (0.53) | 382 (1.19) |
| LTR/ERV1 (%In LTR) | 174 (0.55) | 192 (0.99) | 185 (0.58) |
| LTR/DIRS (%In LTR) | 204 (0.64) | 146 (0.75) | 241 (0.75) |

**Table S6.** Information of function annotation in *Laccaria* genome.

| **Sp.** | **A1(*L. bicolor*) high-altitude** | **B2 (*L. tortilis*) low-altitude** | **C3 (*L. tortilis*) high-altitude** |
|---|---|---|---|
| {NR} % genes | 13989 (58.98) | 18136 (71.73) | 18228 (66.17) |
| {KEGG} % genes | 11902 (50.18) | 17694 (69.98) | 16265 (59.05) |
| {GO} % genes | 9733 (41.03) | 14266 (56.43) | 13433 (48.77) |
| {Pfam} % genes | 9734 (41.04) | 14267 (56.43) | 13434 (48.77) |
| {KOG} % genes | 1675 (7.06) | 2571 (10.17) | 2276 (8.26) |

**Table S7.** CAZymes in *Laccaria* genome.

| **species** | **PL8** | **PL7** | **PL5** | **PL9** | **PL37** | **PL35** | **PL14** | **GT94** | **GT90** | **GT9** | **GT83** | **GT8** | GT76 | GT69 |
|---|---|---|---|---|---|---|---|---|---|---|---|---|---|---|
| A | - | - | - | - | - | - | 2 | - | 4 | - | 3 | 3 | 1 | 3 |
| B | - | - | - | 1 | 1 | 1 | 4 | 1 | 11 | 7 | 6 | 3 | - | 1 |
| C | 1 | 1 | 1 | - | - | 35 | - | - | 5 | 2 | 8 | 4 | 4 | 4 |
| species | GT66 | GT59 | GT58 | GT57 | GT51 | GT5 | GT48 | GT46 | GT4 | GT41 | GT39 | GT35 | GT30 | GT32 |
| A | 1 | 1 | 1 | 1 | 4 | 1 | 2 | 1 | 13 | - | 2 | - | - | 1 |
| B | 2 | - | - | - | 6 | 5 | 2 | - | 37 | 2 | 4 | 4 | 1 | - |
| C | 1 | - | - | - | 8 | 1 | 2 | - | 13 | - | 4 | 1 | 1 | 1 |
| species | GT28 | GT24 | GT22 | GT21 | GT20 | GT2 | GT19 | GT17 | GT15 | GT11 | GT1 | GH99 | GH97 | GH95 |
| A | 1 | 1 | 2 | - | 3 | 23 | - | - | 6 | - | 1 | - | - | - |
| B | 1 | 2 | 2 | 2 | 9 | 51 | 1 | 1 | 3 | 1 | 6 | 2 | 2 | 1 |
| C | 1 | 1 | 3 | - | 1 | 31 | 1 | - | 7 | - | 1 | - | - | 2 |
| species | GH93 | GH92 | GH9 | GH88 | GH87 | GH81 | GH79 | GH78 | GH77 | GH76 | GH73 | GH72 | GH71 | GH65 |
| A | 1 | 3 | 1 | 2 | 1 | 1 | 3 | 1 | - | - | - | 4 | 3 | 2 |

| | | | | | | | | | | | | | | |
|---|---|---|---|---|---|---|---|---|---|---|---|---|---|---|
| B | 1 | 13 | - | 3 | 1 | - | 2 | 7 | - | 6 | 5 | 4 | 5 | 1 |
| C | - | 1 | 2 | 2 | - | 1 | - | - | 2 | - | 1 | 2 | 2 | 1 |
| species | GH64 | GH63 | GH6 | GH55 | GH51 | GH50 | GH5 | GH47 | GH46 | GH43 | GH42 | GH4 | GH39 | GH38 |
| A | - | 1 | 1 | 4 | 2 | - | 18 | 5 | 1 | 1 | 1 | 2 | - | 2 |
| B | 2 | 2 | - | 3 | 2 | - | 30 | 9 | - | 5 | 1 | - | - | 2 |
| C | - | 1 | 3 | 4 | 2 | 1 | 26 | 3 | - | 1 | 1 | 1 | 1 | 2 |
| species | GH37 | GH36 | GH35 | GH33 | GH32 | GH31 | GH30 | GH3 | GH29 | GH28 | GH27 | GH26 | GH25 | GH24 |
| A | 2 | 1 | 3 | - | - | 3 | 2 | 3 | - | - | - | 1 | 2 | 2 |
| B | 2 | 1 | 4 | 1 | 1 | 7 | 4 | 8 | 4 | - | 3 | 1 | 1 | 3 |
| C | 2 | - | 1 | - | - | 3 | 1 | 5 | - | 1 | 1 | 1 | 2 | 2 |
| species | GH23 | GH20 | GH2 | GH19 | GH18 | GH17 | GH163 | GH16 | GH154 | GH152 | GH153 | GH15 | GH146 | GH144 |
| A | - | 1 | 2 | - | 14 | 1 | - | 8 | 1 | 1 | - | 1 | - | - |
| B | 7 | 8 | 12 | 1 | 13 | 3 | 1 | - | 7 | 2 | - | 6 | 2 | 1 |
| C | 4 | 3 | 1 | 1 | 11 | 3 | - | 4 | 1 | - | 2 | 4 | - | - |
| species | GH140 | GH139 | GH133 | GH141 | GH131 | GH130 | GH13 | GH129 | GH128 | GH114 | GH127 | GH125 | GH12 | GH110 |
| A | - | - | - | 2 | 1 | 1 | 20 | - | 9 | 1 | - | - | - | - |
| B | 1 | 1 | 1 | - | - | 2 | 24 | - | 9 | - | 2 | 1 | 2 | 1 |
| C | - | - | - | 2 | 1 | 1 | 20 | 1 | 3 | 2 | - | - | 1 | 1 |
| species | GH109 | GH106 | GH105 | GH102 | GH103 | GH1 | CE9 | CE8 | CE7 | CE5 | CE4 | CE3 | CE16 | CE14 |
| A | 1 | - | - | - | - | 2 | 1 | 1 | 1 | 1 | 2 | - | - | 2 |
| B | 4 | 1 | 9 | - | 1 | 2 | 4 | 2 | - | - | 8 | 1 | 3 | 2 |
| C | - | - | - | 1 | 2 | 1 | 2 | 1 | 2 | 1 | 7 | 1 | 1 | 1 |
| species | CE11 | CE10 | CE1 | CBM9 | CBM67 | CBM6 | CBM57 | CBM48 | CBM44 | CBM38 | CBM35 | CBM32 | CBM16 | CBM21 |
| A | - | 8 | 2 | - | 1 | 1 | - | 2 | 5 | 1 | 1 | 8 | 1 | - |
| B | 2 | 32 | 12 | 1 | 6 | 2 | 3 | 5 | - | - | - | 5 | - | 2 |
| C | 1 | 10 | 5 | - | - | 6 | - | 2 | - | 1 | 1 | 5 | 1 | 1 |
| species | CBM20 | CBM13 | AA9 | AA7 | AA6 | AA5 | AA4 | AA3 | AA2 | AA14 | AA12 | AA1 | | |
| A | - | 1 | 2 | 4 | 1 | 3 | - | 10 | 2 | 2 | - | 7 | | |
| B | 1 | 4 | 4 | 8 | 5 | 7 | 5 | 33 | 6 | 4 | 2 | 16 | | |
| C | 1 | 1 | 3 | 5 | 3 | 1 | 1 | 17 | - | - | - | - | | |
